# Research progress on the effects and mechanisms of different cell types on osteoclast differentiation in bone metabolism in rheumatoid arthritis

**DOI:** 10.3389/fmed.2025.1643285

**Published:** 2025-08-20

**Authors:** Yingjun Wei, Xingwen Xie, Dingpeng Li, Xuan Hou, Ling Ren, Kangwei Wan

**Affiliations:** ^1^Gansu University of Chinese Medicine, Lanzhou, China; ^2^Affiliated Hospital of Gansu University of Chinese Medicine, Lanzhou, China; ^3^Gansu Second People’s Hospital, Lanzhou, China; ^4^Gansu Medical College, Pingliang, China

**Keywords:** OC differentiation, bone metabolism, rheumatoid arthritis, research progress, the effects and mechanisms

## Abstract

Rheumatoid arthritis is a systemic autoimmune disease characterized by chronic synovial inflammation, autoantibody production and progressive joint destruction. One of the main pathological features is irreversible damage and dysfunction of bone and joints, and the core pathological link is osteoclast-mediated imbalance of bone metabolism. With the advances in immunology, molecular biology and cytology, different types of cells, including T cells, B cells, macrophages, natural killer cells, synovial fibroblasts and vascular endothelial cells, activate osteoclasts in rheumatoid arthritis, leading to bone metabolism imbalance in RA and causing bone and joint damage. In this paper, we will systematically summarize the effects and mechanisms of different cell types on osteoclast differentiation in rheumatoid arthritis bone metabolism, which will provide theoretical basis and practical guidance for the precise treatment and targeted intervention of RA bone metabolism abnormalities.

## 1 Introduction

Rheumatoid arthritis (RA) is a complex disease characterized by chronic synovial inflammation, progressive joint destruction, and systemic autoimmune response (1), with a global prevalence of approximately 0.5 to 1% (2). One of the main pathological features leads to joint bone destruction, which seriously affects patients’ joint function and quality of life (3). Actually, osteoclast (OC)-mediated bone erosion is a key link in joint destruction. RA bone metabolism is regulated by a variety of cells, and recent studies have shown that (4, 5), the nature of the abnormalities of RA bone metabolism is the result of the cytokine-mediated regulation of the bone metabolism of immune and non-immune cells. The imbalance of bone metabolism caused by abnormal OC differentiation leads to localized joint bone erosion and destruction. Based on this, this paper will review the roles of different cell types in OC differentiation of RA bone metabolism and their research progress to provide new perspectives for the development of multi-targeted combined therapeutic strategies against RA bone metabolism abnormalities.

## 2 RA and bone metabolism

Bone metabolism is the result of bone remodeling and is mainly regulated by two cell types, OC, which is responsible for bone resorption, and OB, which is responsible for bone formation, and the dynamic balance between the two is important to maintain the normal physiological metabolism and homeostasis of the skeleton. When osteoclastic activity is hyperactivated and osteoblastic function is inhibited, OC activation is increased, causing an imbalanced OC/OB relationship, with the occurrence of abnormal bone meabolism. and the OC/OB relationship is imbalanced, abnormal bone metabolism occurs (6). Both RA and osteoporosis (OP) are closely related diseases of bone metabolism. However, there are significant differences in the activation mechanisms and influence patterns of OC in the two diseases. Them. In OP, the activation of OC is mostly closely related to systemic factors, such as estrogen deficiency and aging, which is manifested as systemic decrease in bone density and bone strength, and the same manifestations occurs in the early period of RA. In RA, the activation of OC is limited to localized joints and interacts with inflammation, forming a complex network of “immunity/inflammation-OC overactivation-bone metabolism imbalance,” which ultimately drives the progressive erosion and destruction of bones and joints in RA patients (6). It has been shown that about 2/5 rheumatoid arthritis patients have bone erosion (7, 8). In RA, the concentration of inflammatory factors, such as TNFα, IL-1, and IL-6, is elevated in the synovium and joints, forming a localized inflammatory microenvironment and activating the activity of OC precursors in the synovium. At the same time, immune cells such as Th17 cells and B cells and synovial fibroblasts in the synovium activate OC by secreting pro-inflammatory TNF-*α*, IL-17 and other cytokines and signaling molecules such as RANKL, and activated OC releases catalases and acid enzymes, which are the main causes of increased bone resorption leading to bone erosion in RA patients (9). Elevated levels of inflammatory cytokines inhibit bone formation not only by activating OC but also by inhibiting OB activity. Multiple pathologic mechanisms intersect with each other eventually causing joint and bone destruction and joint deformity (10). The current first-line therapeutic drugs for RA include methotrexate (MTX) and glucocorticoids, etc. However, studies have shown that MTX can prevent OB reduction and increased bone resorption. Long-term use of glucocorticoids will inhibit the activity of osteoblasts and simultaneously activate the OC precursors in the bone marrow to promote bone resorption, which affects bone metabolism and causes a decrease in bone density (3). In addition, several studies have found (11, 12) that vitamin D deficiency is negatively correlated with disease activity in RA, and that vitamin D supplementation significantly improves disease activity in RA patients. 1,25-dihydroxyvitamin D3, the active form of vitamin D, can regulate calcium metabolism, reduce parathyroid hormone secretion, maintais bone metabolism homeostasis, and have an immunomodulatory effect that reduces the production of pro-inflammatory cytokines (13). Therefore, for the abnormal bone metabolism in RA, in addition to conventional anti-inflammatory treatment, attention needs to be paid to the regulatory mechanisms of OC to achieve more effective bone protection.

## 3 Osteoclast differentiation and RA bone metabolism

OC is the only multinucleated giant cell with bone resorption function *in vivo* and plays a crucial role in maintaining bone homeostasis. OB is differentiated from MSCs. It stimulates the differentiation of OC precursors through secreting regulatory factors, such as nuclear factor κB receptor-activating factor ligand (RANKL) and macrophage colony-stimulating factor (M-CSF), meanwhile, inhibits overactivation involved in the regulation of OC differentiation by secreting OPG (RANKL decoy receptor) (14, 15). In physiological state, OC-mediated bone resorption and OB-dominated bone formation maintain a dynamic balance and work together to maintain skeletal metabolic homeostasis. However, under RA pathology, abnormal expression of RANKL and M-CSF occurs, which in turn disrupts the original equilibrium and significantly promotes OC differentiation and activation. In RA patients, the levels of pro-inflammatory cytokines, such as TNF-*α* and IL-6, are significantly elevated, inducing an increase in the expression of RANKL in peripheral blood mononuclear cells (PBMC) (16), and the high expression of RANKL drives the generation of a large number of highly activated OC, which increases bone resorption, and is the core driver of the imbalance of bone metabolism and bone destruction in RA patients (17). Studies have shown (18) that the accumulation of large amounts of OC in synovial tissues of RA patients, and the interaction between bone metabolic imbalance caused by abnormally differentiated OC and the local inflammatory microenvironment are the main causes of intra-articular bone erosion and localized bone destruction in the joints of RA patients. OC also plays an important role in the regulation of the immune system (19). In the RA pathological environment, OC actively secretes factors such as TNF-*α*, IL-6, and IL-1 during bone resorption, which affects the activity of immune cells and drives macrophage polarization toward M1 type (20). It has been found that macrophages are circulating precursors of OC, and synovial macrophages can develop into mature OC with luminal absorptive capacity, through which forming a self-perpetuating cycle of “OC-inflammatory factor-M1 macrophage-new OC” (21). The over-activation of OC has an important impact on the RA immune microenvironment. In addition, RA synovial tissue maintains a hypoxic microenvironment due to vascular opacification, which induces stable expression of hypoxia-inducible factor 1α (HIF-1α) and up-regulation of RANKL, and further promotes differentiation of osteoclastic precursor cells into OC (22, 23). Calcium ions (Ca^2+^) released by mature OC can activate RA synovial fibroblasts (RA-FLSs), and activated RA-FLSs can secrete more M-CSF and inflammatory factors, which will further enhance the bone resorption activity of OC, and release more Ca^2+^ by differentiating matured OC. In reverse, the increasing Ca^2+^ will stimulate RA-FLSs to secret more related factors to exacerbate the imbalance of RA bone metabolism and aggravate the condition, Such a vicious circle ultimately leads to the thorough disruption of bone metabolism balance (24) and makes the degree of erosion and destruction of RA patients’ bones and joints more and more serious. The activation and differentiation of OC not only lead to bone resorption, but also degrade the bone matrix through the release of a variety of enzymes such as TRAP, CTSK and MMP9, results in further exacerbation of joint destruction. What’s more, it dynamically interacts with the inflammatory and hypoxic microenvironment of the synovial tissues, which ultimately drives the progressive erosion and destruction of the bones and joints of patients with RA, and weave together the complex network of “immunity/inflammation - over-activation of OC - imbalance of bone metabolism - bone destruction of joints.

## 4 Effect of different cell types on osteoclast differentiation of bone metabolism in rheumatoid arthritis

**Figure 1:**
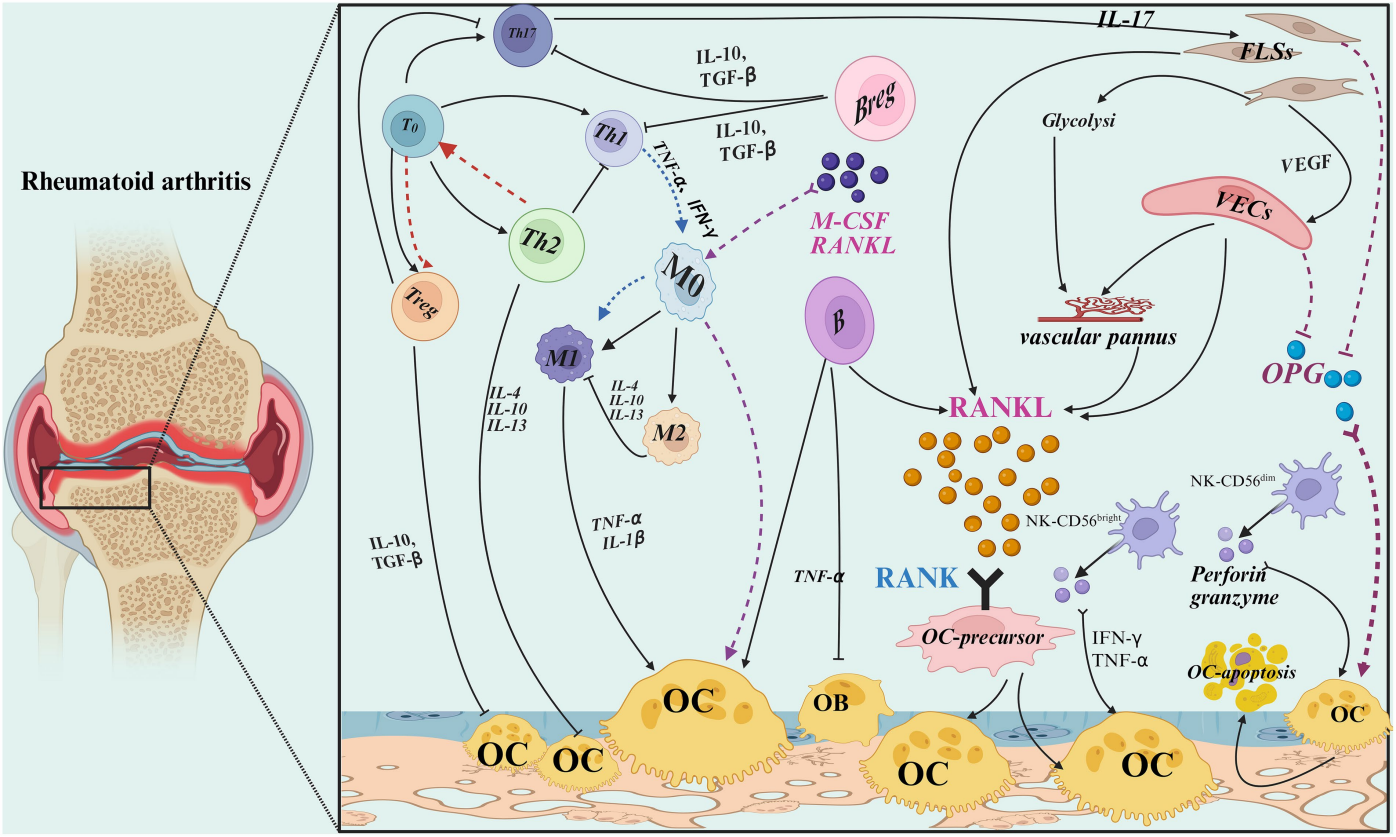
Effect of different cell types on OC differentiation of rheumatoid arthritis bone metabolism [Created with BioRender (https://app.biorender.com/)].

OC is the driving and critical cells for bone erosion and destruction in RA. The differentiation of OC in RA can be influenced by different cell types, and immune and non-immune cells form a complex regulatory network by secreting different cytokines and regulating metabolic reprogramming, ultimately leading to over-activation of OC and imbalance of bone resorption (Table 1; Figure 1).

**Table 1:** Effects of different cell types on osteoclast differentiation of bone metabolism in rheumatoid arthritis*.

Cell type	Subtype	Effect on OC differentiation	Key effector molecules/pathways	Localization and characterization in RA bone erosion	Reference
T-cells	Th17	Significantly promote	IL-17, RANKL↑, OPG↓	Localized secretion of IL-17 in synovium → activation of FLSs-OC axis; maintenance of immune homeostasis with Treg	(17)
	Treg	Inhibit	TGF-*β*, IL-10	Decreased quantity, functional exhaustion → weakened inhibition of Th17/OC	(30)
	Th1	Promote	TNF-α, IFN-γ	Enhance OC differentiation via TNFR1; induces M1 macrophagy	(35) Th2
	Th2	Inhibit	IL-4, IL-13	Inhibited function → diminished negative regulation of Th1	(8)
B-cells	B cells	Promote	TNF-α, RANKL, ACPA	Produce autoantibodies + proinflammatory factors; memory B High expression of RANKL	(38, 39)
	Breg	Inhibit	IL-10, TGF-β	Defective quantity or function → insufficient bone protective signaling	(41)
Macrophages	M1	Significantly promote	TNF-α, IL-1β, RANKL	Homologous to OC precursors; creates “M1-OC” positive feedback	(7, 8, 35)
	M2	Inhibit	IL-10, TGF-β	Anti-inflammatory repair phenotype	(42)
NK-cells	CD56^dim^	Inhibition	Perforin/granzyme	Peripheral blood predominant; induces OC apoptosis → inhibits bone destruction	(44)
	CD56^bright^	Promote	IFN-γ, TNF-α	Synovial infiltration → drives M1/Th17 polarization	(45)
FLSs	RA-FLSs	Significantly promote	RANKL↑, OPG↓, MMPs, VEGF	Localized synovial “central amplifier”; hypoxia-glycolytic reprogramming; induces vascular; RANKL source → enhanced OC differentiation	(48, 50, 54, 57, 58)
VECs	VECs	Promote	VEGF, RANKL ↑, OPG ↓	Synovial vascular pannus endothelium; hypoxia-VEGF axis drive OC recruitment and activation	(60–62)

*Th17, T helper 17 cells; Treg, Regulatory T cells; Th1, T helper 1 cells; Th2, T helper 2 cells; Breg, Regulatory B cells; NK Cells, Natural killer cells; FLSs, Fibroblast-Like Synoviocytes; VECs, Vascular Endothelial Cells; RA-FLSs, Rheumatoid Arthritis Fibroblast-Like Synoviocytes.

### 4.1 T cells

CD4^+^ T cell is one of the important T cell subpopulations that can differentiate into subpopulations of pro-inflammatory Th1 and Th17 cells as well as Th2 and Treg cells with immunomodulatory functions (25). Studies have shown (26, 27) that dysregulation of the ratios of subpopulations such as Th1/Th2 and Th17/Treg can cause abnormalities in bone metabolism, which collectively drive ongoing autoimmune responses and osteoarticular destructive injury.

#### 4.1.1 Th17/Treg cells

Under physiological conditions, Th17 and Treg cells constrain each other and work together to maintain the dynamic balance of bone metabolism. In RA, Th17/Treg imbalance is one of the drivers of abnormal bone metabolism (28). In the hypoxic synovial microenvironment, IL-17 secreted by Th17 cells activates the NF-κB pathway of FLSs, upregulates RANKL expression and inhibits OPG secretion, and promotes the differentiation of OC precursor cells into mature OC (17). In addition, Th17 cells activate FLSs and macrophages to release inflammatory mediators such as TNF-*α*, IL-1*β*, and IL-6, which enhance OC activity and lead to irreversible destruction of articular cartilage and bone tissues (29). Treg cells inhibit osteoclastic activity by suppressing overactivation of Th17 cells and secretion of TGF-β (30). Defective Treg cell function leads to uncontrolled osteoclastic activity and exacerbates abnormal OC activation (31). In RA patients, Treg cells are reduced in number and impaired in function, and IL-6 in the inflammatory microenvironment further inhibits the expression of the Treg cell transcription factor Foxp3, which promotes the differentiation of Th17 cells and impairs their inhibitory effects on OC production and bone resorption (32, 33).

Therefore, therapeutic strategies targeting the Th17/Treg axis (e.g., IL-17 inhibitors, Treg cell relay therapy) can not only alleviate inflammation, but also directly inhibit articular bone erosion through dual modulation of immune homeostasis and OC activation. However, how to overcome the functional depletion of Treg cells in the RA inflammatory microenvironment remains a key challenge for clinical translation, and has become an important research direction for the treatment of RA bone metabolic abnormalities with potential clinical applications.

#### 4.1.2 Th1/Th2 cells

In RA patients, pro-inflammatory Th1 cells are activated and the function of Th2 cells is suppressed (34). The imbalance in the Th1/Th2 ratio is closely associated with the development of RA bone metabolism abnormalities (26). TNF-*α*, a pro-inflammatory cytokine secreted by Th1 cells can bind to tumor necrosis factor receptor 1 (TNFR1), so that the involvement of TNFR1 makes bone resorption promote bone destruction through the enhancement of OC differentiation, thereby exacerbating joint damage (35). In addition, T-bet, a key transcription factor of Th1, not only enhances the self-polarization of Th1 cells by promoting the secretion of pro-inflammatory factors such as IFN-*γ*, but also induces macrophage polarization toward M1 type and promotes neutrophil inflammatory infiltration. Together, these effects lead to localized pathological changes such as cartilage destruction and bone erosion in the joints (36). Th2 cells negatively regulate the functional activity of Th1 cells. Th2 cells secrete the OC inhibitory cytokines IL-4, IL-10, and IL-13 to inhibit OC formation (8), and the relative insufficiency of Th2 cells and the overactivity of Th1 cells lead to ineffective control of inflammatory response, thereby accelerating the disease progression of RA bone and joint destruction (37).

Based on the above mentioned mechanisms, the current study proposes a new strategy to treat RA by regulating the Th1/Th2 balance: inhibiting the overactivation of Th1 cells and promoting the function of Th2 cells, thereby restoring bone metabolic homeostasis. This strategy will provide a new target for intervention in the treatment of RA.

### 4.2 B cells

B cells in the peripheral blood of RA patients can secrete a variety of different cytokines and participate in bone metabolism through multiple mechanisms. B cells promote OC formation by secreting the pro-inflammatory cytokine TNF-*α* and increasing RANKL expression, meanwhile inhibiting osteoblast differentiation, leading to bone erosion and joint destruction (38). In addition, anti-citrullinated protein antibody (ACPA) produced by B cells activates immune cells, upregulates pro-inflammatory cytokines, and induces extracellular trapping of neutrophils, which further promotes OC differentiation and affects bone metabolism (39). B cells also directly act on the RANKL/RANK/OPG signaling pathway, regulate the balance of cytokines related to OC differentiation, and participate in bone metabolism processes (40). Regulatory B (Breg) cell is a type of B cells that exert immunosuppressive functions. They can secrete and produce anti-inflammatory factors such as TGF-*β* and IL-10, inhibit pro-inflammatory Th1 and Th17 cells, reduce the production of inflammatory factors such as IFN-*γ*, IL-17, IL-21, and induce the differentiation of the initial CD4^+^ T cells to Treg, which can inhibit the osteoclasts differentiation directly or indirectly and reduce bone destruction (41).

In summary, B cells have an important role in the differentiation of OC in bone metabolism in RA, and this complex biology makes them a potential target for RA therapy. At present, targeted therapy against B cells has shown good results in clinical applications, but how to selectively regulate different functional subpopulations of B cells remains to be further investigated.

### 4.3 Macrophages

Depending on the microenvironment, macrophages can be polarized into functionally distinct subpopulations of M1-type and M2-type. M2-type macrophages have significant anti-inflammatory and tissue-repairing functions, participating in processes such as tissue remodeling and inflammation suppression, and their immune-tolerance properties help to limit excessive inflammatory damage (42). In contrast, M1-type macrophages lose their tolerance in the pro-inflammatory microenvironment, and directly or indirectly affect bone metabolic homeostasis by secreting large amounts of pro-inflammatory cytokines, activating RANKL, and promoting OC activation to promote OC differentiation while inhibiting OB differentiation (21, 43). In addition, osteoclast precursor cells share a common origin with macrophages. OCs are usually derived from the monocyte/macrophage lineage, which differentiate into OC in response to M-CSF and RANKL, forming a “macrophage - OC” positive feedback loop that promotes OC production and activation. OC overproduction or increased activity enhances OC bone resorption and exacerbates articular cartilage and bone destruction (20). Metabolic reprogramming is also an important factor affecting macrophage polarization. Metabolically reprogrammed M1-type macrophages also secrete matrix metalloproteinases and chemokines, recruit neutrophils and promote cartilage matrix degradation, accelerating the destruction of joint structures (44).

It is summarized. The polarizaiton sate and metabolic reprogramming of macrophages that macrophage polarization status and metabolic reprogramming affect OC differentiation and directly influence bone destruction in RA. By regulating polarization and metabolic pathways of macrophage, new strategies could be provided for the treatment of RA. Future therapies may focus on targeting specific metabolic pathways and signaling pathways in macrophages to achieve more effective therapeutic effects.

### 4.4 Natural killer cells

Playing an important role in the pathogenesis of autoimmune diseases such as RA, there is an abnormal infiltration of natural killer (NK) cells in synovial tissue and synovial fluid of RA patients (45). According to the expression level of CD56, the surface marker, NK cells can be classified into two main subpopulations, CD56^dim^ and CD56^bright^ (46). CD56^dim^ NK cells subpopulation accounts for about 90% of the NK cells in peripheral blood, which mainly exist in the blood, and can play a protective role in inhibiting bone destruction by releasing perforins and granzymes, which induces apoptosis of OC. While CD56^bright^ NK cells are mainly found in the peripheral inflammatory sites of lymph nodes, and can secrete and produce inflammatory cytokines such as IFN-*γ*, TNF-*α*, etc. IFN-γ can induce macrophage M1 polarization and Th17 inflammation-promoting cell differentiation (47), which promotes the generation of OC and further exacerbates the osteoarthritic injury response, thus promoting the process of RA. TNF-α can induce OC differentiation causing cartilage and bone destruction. Together, these above mentioned mechanisms exacerbate inflammation and joint destruction in RA (48).

Taken together, NK cells have a complex dual role in RA bone metabolism, either inhibiting OC through cytotoxic effects or potentially exacerbating OC differentiation through cytokine secretion and immunomodulation. These findings provide new potential targets for the treatment of RA.

### 4.5 Fibroblast-like synoviocytes

FLSs are important constituent cells of synovial tissue, They are involved in cartilage nutrient supply and joint surface lubrication by secreting extracellular matrix (ECM) components and joint lubricating substances, which are critical for the maintainance of joint collagen homeostasis (49). Under normal physiological conditions, the synovial microenvironment maintains a dynamic balance through the development, differentiation and apoptosis of FLSs. In RA, FLSs activates and differentiates into RA-FLSs with an aggressive phenotype, which is one of the effector cells of RA bone metabolism abnormality (50). The pro-inflammatory cytokines, such as TNF-*α*, IL-1β, and IL-6, as well as the chemokines, such as CXCL12 and CCL2, which are abnormally secreted by RA-FLSs, contribute a complex inflammatory network, can not only attract the migration of OC precursors to migrate toward the site of inflammation and promote their differentiation into mature OC, but also regulate OC migration, to form a positive feedback loop that exacerbates bone destruction through its interaction with inflammatory cells (51). RA-FLSs can secrete matrix metalloproteinases (MMPs) capable of degrading the cartilage matrix on the articular surface, disrupting the structural integrity of the bone tissue and creating favorable conditions for OC invasion and bone resorption (52).

RANKL is a major regulator of OC differentiation (53), while RA-FLSs is an important source of RANKL in the synovium of RA (54, 55). RA-FLSs indirectly induces osteoblasts to secrete RANKL through cytokine networks, and triggers the differentiation of OC by directly binding to the OC precursor RANK receptor. At the same time, RA-FLSs inhibits the secretion of osteoprotegerin (OPG), disrupts the RANKL/OPG balance, further promotes OC production and inhibits the bone repair effect, and inhibits the formation of osteoblasts under inflammatory conditions, which is the core effector cell of RA bone and joint destruction and the “central amplifier^”^ in the process of bone destruction (50, 52, 56). In addition, over-activated RA-FLSs promotes the stratification and stacking of FLSs to form inflammatory granulation tissue, called vascular pannus (57). Vascular opacities can produce large amounts of RANKL, which promotes OC differentiation leading to osteoclastic and articular destruction (58).

RA-FLSs are the core target cells of RA synovium, and the excessive proliferation and differentiation of RA-FLSs further aggravate the hypoxic state of synovial tissue, and the hypoxic microenvironment of synovium provides favorable conditions for OC generation. Researches show that hypoxia down-regulation of copper metabolism MURR1 structural domain (COMMD1) protein expression increases RANKL-induced inflammation as well as downstream OC production, resulting in bone destruction (59). In the synovial hypoxic microenvironment of RA, the energy metabolism of RA FLSs changes, manifested as the initiation of glucose metabolism reprogramming: Shifting from aerobic respiration to anaerobic glycolysis, the enhanced aerobic glycolysis exhibited by RA-FLSs not only provides energy and biosynthetic precursors for its rapid proliferation and promotes the invasive ability and vascular pannus formation of RA-FLSs, but also remodels the articular microenvironment through mechanisms such as lactic acid buildup, which further induces osteoclasts differentiation and migration, and accelerates the process of articular cartilage destruction and degeneration (60, 61).

In summary, RA-FLSs exhibits abnormal proliferation, migration and invasive ability, and secretes a large number of pro-inflammatory cytokines and chemokines, which form a bidirectional feedback loop by regulating RANKL expression, metabolic reprogramming and other mechanisms, driving the abnormal activation of OC, which collectively disrupts the balance of bone metabolism and ultimately leads to bone erosion and joint destruction in RA patients. The above pathologic processes play an important role in the pathogenesis of RA and provide multiple potential targets for intervention in the clinical treatment of RA.

### 4.6 Vascular endothelial cells

VECs, located at the vascular-tissue interface, play a crucial role in the regulation of bone metabolism. They not only constitute the basic structure of the vascular wall, but also directly or indirectly influence the activation and differentiation of OC through multiple biological pathways like inflammatory responses, angiogenesis and cytokine secretion, which in turn participate in the pathological process of bone destruction (62). In the pathological environment of RA, RA synovial hypoxia induces endothelial cells to highly express VEGF, which binds to VEGFR receptors on VECs and stimulates VECs to proliferate and migrate to stimulate (63). Abnormal angiogenesis activates OC and releases proteases, which are mutually reinforcing with inflammatory cell invasion, forming a vicious cycle, exacerbating joint inflammation and destruction, and accelerating the damage of cartilage and bone. Thereby exacerbating the pathologic process of RA (64).

In addition, VECs maintain bone metabolism homeostasis by simultaneously secreting RANKL and OPG through paracrine effects, and this homeostasis is essential for maintaining the stability of bone metabolism. Under normal physiological conditions, VECs usually inhibit OC differentiation by secreting OPG, thus protecting bone (65). However, in RA patients, due to the inflammatory response, immune dysregulation, and hypoxic microenvironment, activated VECs abnormally secrete RANKL and OPG, which further amplifies osteoclastic signals and promotes OC formation and activation, leading to excessive bone resorption and destruction (17).

In a word, the role of VECs in RA bone destruction goes far beyond being a source of inflammatory factors; they are important regulators of OC differentiation. By secreting multiple bioactive factors, VECs act synergistically to jointly promote OC differentiation and functional activation. An in-depth understanding of these mechanisms not only helps to reveal the pathological nature of bone destruction in RA, but also provides potential therapeutic targets for the development of intervention strategies against RA bone destruction.

## 5 Summary and outlook

The mechanism of bone metabolism malfunction in RA is a complex pathological process involving multiple immune cells and mesenchymal stromal cells, the core of which lies in the disruption of bone metabolism homeostasis caused by the chronic inflammatory microenvironment triggered by immune system dysregulation. Under normal physiological conditions, the dynamic balance of bone metabolism whose function is strictly regulated, relies on the synergistic action of multiple cells regulating OC differentiation. However, in the pathological environment of RA, multiple pro-inflammatory factors and mediators interact with each other in the microenvironment of inflammation, hypoxia, and immune dysregulation, which significantly promotes the abnormal activation and differentiation of OC, exacerbates bone erosion and the destruction of joint structure, and eventually leads to irreversible bone damage and dysfunction in RA patients.

Among the immune abnormalities in RA, the aberrant interactions between T cells and B cells are particularly critical. The binding of T cells to CD40 on the surface of B cells through surface CD40L not only activates signaling pathways within B cells to promote their proliferation and differentiation, but also further strengthens the cellular immune response through the recognition of B-cell MHC-antigen peptide complex by the T-cell receptor (TCR) (66, 67). B cells present citrullinated antigenic peptides to T cells via MHC-II, activating autoreactive CD4^+^ T cells, especially Th1 and Th17, which directly stimulate OC differentiation. In addition, although B cells secrete OPG to maintain bone metabolic homeostasis (68), their abnormally activated cells secrete RANKL to promote OC formation. Thus, a vicious cycle of “T-B cell contact → B cell activation → autoantibodies + proinflammatory factors → synovial inflammation → bone destruction” is formed. Macrophages in RA synovium show a significant M1/M2 polarization imbalance, with M1-type macrophages predominating and releasing large amounts of pro-inflammatory factors, such as TNF-*α*, IL-1β, IL-6, etc. These cytokines not only directly stimulate OC precursor cell differentiation, but also activate FLSs and promote their secretion of RANKL and MMPs, which further exacerbate bone resorption and cartilage degradation. What is more, M1-type macrophages maintain a chronic inflammatory state through the NF-κB and MAPK signaling pathways, forming a positive feedback loop that perpetuates the progression of bone destruction. NETs cause joints damage by activating RA-FLSs. By delivering the citrullinated peptide segments that they carry to FLSs, NETs not only activate the antigen-presenting function of FLSs, but also promote autoimmune responses and cartilage degradation (69). FLSs interacts with VECs through adhesion molecules such as ICAM-1/VCAM-1-integrin, which promotes inflammatory cell extravasation and neovascularization to form vascular opacities. This aberrant interaction also leads to dysfunction of VECs, releasing pro-angiogenic factors such as VEGF and ANG-2, exacerbating synovial hypoxia and acidification, and further stimulating OC activation. Meanwhile, the continuous interaction between FLSs and VECs maintains the chronic inflammatory state of RA, which makes the bone erosion around synovial joints much worse (70). As the disease progresses, the sustained inflammatory response will ultimately lead to bone erosion, joint deformity and loss of function. Bone erosion in RA is the result of multiple cellular interactions: in the initiation phase, the interaction is shown as “abnormal T-B cell contact → autoantibody + Th17 activation → synovial inflammation”; and in the amplification phase, it is expressed like “macrophage M1 polarization, FLSs activation, NETs release → TNF-*α*/IL-6/IL-17 storm→RANKL/OPG imbalance”; while in the Terminal phase, it is manifested as “hypoxic microenvironment + MMPs + OC overactivation→cartilage degradation and bone resorption”.

The abnormal RA bone metabolism is the result of a ternary interaction model of “inflammation/immunity-bone metabolism malfunction-bone erosion and destruction,” which is formed by the interaction of inflammatory immune cell polarization, metabolic reprogramming and bone remodeling signals. However, there still exist several problems in the current research. In the RA-related research, despite the breakthrough progress in exploring the functions of cell types, there are still many key scientific problems in the cell–cell interaction network that need to be solved. At the level of cellular interactions, although it has been confirmed that there is a complex regulatory relationship between various immune cells and mesenchymal stromal cells, the specific molecular mechanisms and signaling pathway networks have not yet formed a systematic knowledge. In addition, cell interactions are dynamically regulated by multi-dimensional factors such as genetic polymorphisms, environmental triggers and immune homeostatic imbalance, and how these factors affect RA disease heterogeneity by remodeling cellular communication networks will be an important direction for future research. An integration of single-cell multi-omics (transcriptome, epigenome) and spatial metabolomic technologies will be required in the future research, so that the spatiotemporal dynamics of immune-mesenchymal cell interactions in RA synovium could be analyzed, and the targeting of specific metabolic pathways could be validated, providing a basis for the development of precision therapies targeting specific cell subpopulations.

## 6 Conclusion

In general, RA, as a complex chronic inflammatory autoimmune disease, has a complex regulation at the cellular level at the core of its pathogenesis. RA bone metabolism is closely related to osteoclasts, and different types of effector cells, including T cells, B cells, and so on, have a significant impact on osteoclast differentiation in RA patients. However, the heterogeneity of interactions between different cell types, cytokine networks and signaling pathways makes individualized treatment a great challenge. Further integrate multidisciplinary techniques will be needed in the future studies to facilitate the development of precision medicine and bring more effective treatment options to patients!
